# Pulsed Laser Beam Welding of Pd_43_Cu_27_Ni_10_P_20_ Bulk Metallic Glass

**DOI:** 10.1038/s41598-017-08460-6

**Published:** 2017-08-11

**Authors:** Ling Shao, Amit Datye, Jiankang Huang, Jittisa Ketkaew, Sung Woo Sohn, Shaofan Zhao, Sujun Wu, Yuming Zhang, Udo D. Schwarz, Jan Schroers

**Affiliations:** 1 Department of Mechanical Engineering and Materials Science, Yale University, New Haven, CT 06511 USA; 2 School of Materials Science and Engineering, Beihang University, Beijing, 100191 China; 3 Department of Mechanical Engineering and Center for Research on Interface Structures and Phenomena (CRISP), Yale University, New Haven, CT 06511 USA; 4 State Key Laboratory of Advanced Processing and Recycling of Non-Ferrous Metals, Lanzhou University of Technology, Lanzhou, 730050 China; 5 Department of Electrical and Computer Engineering and Institute of Sustainable Manufacturing, University of Kentucky, Lexington, KY 40506 USA

## Abstract

We used pulsed laser beam welding method to join Pd_43_Cu_27_Ni_10_P_20_ (at.%) bulk metallic glass and characterized the properties of the joint. Fusion zone and heat-affected zone in the weld joint can be maintained completely amorphous as confirmed by X-ray diffraction and differential scanning calorimetry. No visible defects were observed in the weld joint. Nanoindentation and bend tests were carried out to determine the mechanical properties of the weld joint. Fusion zone and heat-affected zone exhibit very similar elastic moduli and hardness when compared to the base material, and the weld joint shows high ductility in bending which is accomplished through the operation of multiple shear bands. Our results reveal that pulsed laser beam welding under appropriate processing parameters provides a practical viable method to join bulk metallic glasses.

## Introduction

Bulk metallic glasses (BMGs), with their amorphous structure possessing attractive properties, such as high strength and elasticity, which is often coupled with high corrosion resistance and toughness^1–3^. Motivated by these properties and the potential to process them like thermo-plastics, BMGs are currently at the frontier of metals research^4–6^.

One important requirement for any material class to be of practical use for structural applications is the ability to join them to like and dislike materials. Particularly for BMGs, the metastable nature and resulting incompatibility with conventional processing and joining methods have become stumbling blocks for their practical applications^7, 8^. To address the ability to join BMGs, various techniques that have been developed specifically or tailored for BMGs based on liquid-state processing including electron beam welding^9, 10^, laser beam welding^11, 12^, gas tungsten arc (GTA) welding^13^, and pulse current method^14, 15^; or solid-state processing including friction welding^16, 17^, explosion welding^18^, ultrasonic welding^19, 20^, diffusion bonding^21^, spark welding^22^, and resistance spot welding^23^. Recently, a thermoplastic-based method^24^ and a liquid-solid joining method^7^ were introduced. The most important issue in the welding of BMGs is the avoidance of crystallization in the fusion zone (FZ) and heat-affected zone (HAZ), which requires rapid cooling and has been generally challenging for developed joining methods^25, 26^. This is particularly the case for joining methods where the joint region is melted, so the weld must be subsequently cooled fast enough to avoid crystallization^27^. Laser welding can reduce the possibility of crystallization^28–31^ since it results in a deep and narrow weld region with higher cooling rates that can be achieved^32, 33^. Avoiding crystallization in a weld joint of BMG is in general difficult and has only been realized for a small number of BMG formers with high glass forming ability under highly optimized welding parameters^34, 35^.

To address the requirements for the fast cooling we use pulsed laser beam welding method which provides high welding energy concentrated within a narrow zone, and a much shorter retention time, which results in higher cooling rates. The pulsed laser beam welding method was used to do bead-on-plate (BOP) experiments of Pd_43_Cu_27_Ni_10_P_20_ (at.%) BMG under different welding parameters. The weld seams were examined for their amorphous nature. We then used one of the welding parameters of BOP tests to join Pd_43_Cu_27_Ni_10_P_20_ BMG of 1 mm thickness. After joining, the amorphous nature and mechanical properties of the weld joint were investigated. We show that the pulsed laser beam welding method yields precise welds with bulk like mechanical properties.

## Materials and Methods

Pd_43_Cu_27_Ni_10_P_20_ master alloy ingots were prepared by arc-melting a mixture of the high-purity (min. 99.95%) elements in a pure titanium-gettered argon atmosphere with a low oxygen level of 350 ppm. The amorphous state was achieved by rapid quenching of alloy casting-suction into a copper mould. Subsequently, thermoplastic forming (TPF)^36–39^ was used to essentially eliminate casting induced porosity. The amorphous nature of the BMG plates was determined by X-ray diffraction (XRD, Rigaku SmartLab, using Cu Kα radiation), and differential scanning calorimetry (Perkin Elmer, Diamond DSC) at a heating rate of 20 K min^−1^. First, the BOP experiments on Pd_43_Cu_27_Ni_10_P_20_ BMG were carried out using the pulsed laser beam welding setup (IPG PHOTONICS 1500) with 1500 W maximum laser beam power in the wavelength region of 1060–1100 nm. Table 1 shows the welding parameters for the bead-on-plate experiments. Effective peak power density (*EPPD*) determines interaction intensity of laser beam with the BMG for a given spot size considering the pulse overlap (*PO*) during welding process and is given by^40^,

1 $$EPPD=\Gamma \times PPD,$$

where *Γ* is the pulse overlapping index and *PPD* is the peak power density. Due to *Γ* = 1/(1 − *PO*) and *PPD* = *P*_*peak*_/[π(*d*/2)^2^], where *P*_*peak*_ is the peak power of the laser beam, and *d* is the spot diameter, Eq. (1) can be rewritten as,

2 $$EPPD=\frac{{P}_{peak}}{\pi {(\frac{d}{2})}^{2}(1-PO)}$$

where *PO* = 1 − *ν*/*df*, *ν* is the welding speed, and *f* is the pulse frequency.

**Table 1:** Welding parameters of the pulsed laser beam welding.

Peak power, *P*_*peak*_ (W)	Duty cycle, *D* (%)	Welding speed, *ν* (mmmin^−1^)	Pulse frequency, *f* (Hz)	Spot diameter, *d* (mm)
750	7	20	10	1
825	7	20	10	1
900	7	20	10	1
975	7	20	10	1
1050	7	20	10	1
1125	7	20	10	1

After welding, the appearance of the weld seam was observed with a VHX-500F digital optical microscope (OM). Samples were cut perpendicular to the welding direction of the weld joint for characterization.

**Figure 1:**
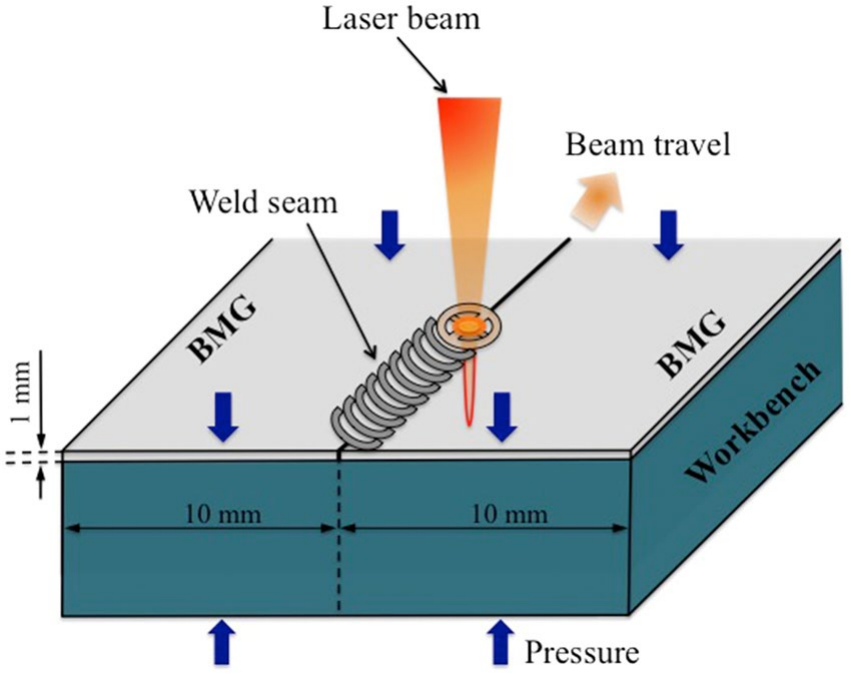
Schematic of the pulsed laser butt welding. A laser beam of controllable power was used to weld metallic glass pieces. The orange arrow indicates the direction of the laser beam is travelling.

**Figure 2:**
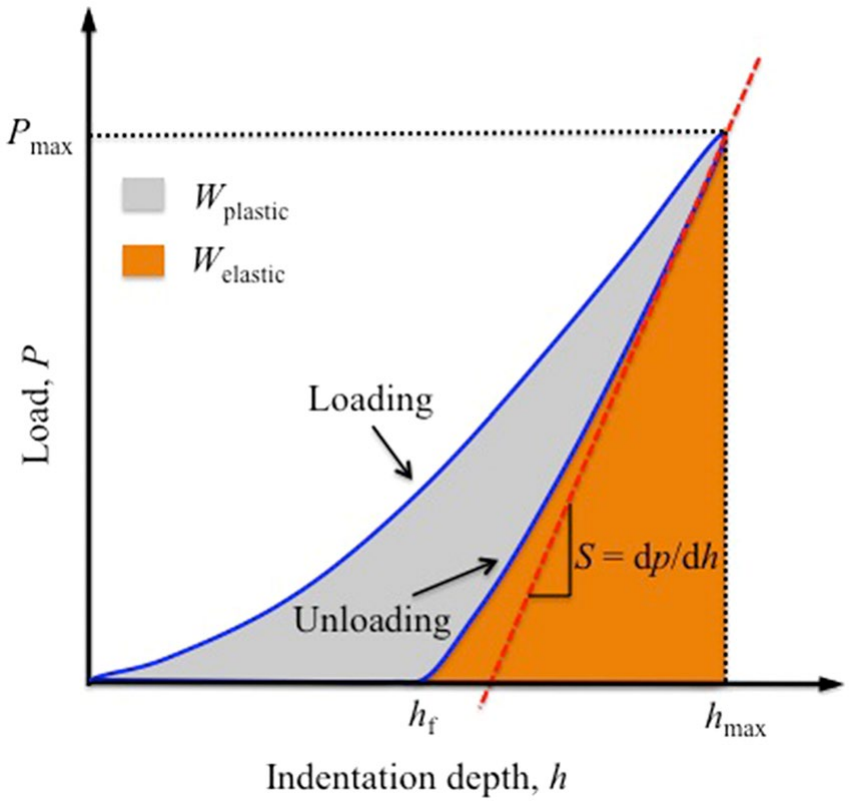
Schematic *P*-*h* curve for Berkovich indentation. *P*_max_ is the maximum indentation load, *h*_max_ is the maximum indentation depth, *h*_f_ is the final depth, the elastic strain energy *W*_elastic_ is the area under the unloading curve, and the indentation absorbed energy *W*_plastic_ is the area under the loading curve.

Subsequently, in order to study the mechanical properties of the weld joint two sheets of Pd_43_Cu_27_Ni_10_P_20_ BMG were joined together using the pulsed laser beam welding method, also called butt welding (Fig. 1). The different zones of FZ, HAZ and base material (BM) in the butt joint were separated in order to further determine their glassy nature. The butt joint of Pd_43_Cu_27_Ni_10_P_20_ BMG was ground on the planar-section of the weld seam with wet abrasive papers and mechanically polished to obtain a mirror-polished test planar-section. In order to characterize the mechanical properties of the butt joint, room-temperature nanoindentation tests were performed on the polished planar-section using a MTS Nanoindenter XP (MTS, Oak Ridge, TN) with a Berkovich tip. All nanoindentation experiments were done using the CSM (Continuous Stiffness Measurement) technique^41^ which gives the load on the sample and the contact stiffness as a function of the displacement of the indenter into the samples. A fused silica specimen with a known modulus was used to calibrate the system. The first indent is 1.5 mm far away from the edge of the butt joint starting at the middle of the weld seam covering a distance of 5 mm. Indentations were spaced at 500 μm along the x-direction and 500 μm along the y-direction. A series of indentations were carried out in a 10 × 4 grid. A specially developed method^42, 43^ was used, which uses exponential loading at a constant strain rate of 0.05 s^−1^, corrects for thermal drift and calculates the average for the modulus and hardness from a penetration depth of 150 nm to 500 nm. All the indentations are up to a peak load of 50 mN. The output of an instrumented indentation test is the load-displacement curve during loading and unloading of the indenter, as shown in Fig. 2 ^44^. *P*_max_ is the maximum indentation load, *h*_max_ is the maximum indentation depth, *h*_f_ is the final depth, the elastic strain energy *W*_elastic_ is the area under the unloading curve (this area represents the elastic energy associated with residual stresses caused by indenter withdrawal), and the indentation absorbed energy *W*_plastic_ is the area under the loading curve (this area represents the energy dissipated during indentation due to plastic deformation, cracking and crushing processes).

In Fig. 2, the loading portion of the load-displacement curve is often described by Meyer’s law^45^,

3 $$P={k}_{1}{h}^{n}$$

where *P* is the instantaneous load, *k*_1_ is the loading curve constant, *n* is the loading exponent and *h* is the instantaneous depth. The total energy (characterizing energy-absorbing or energy-releasing events occurring beneath an indenter), *W*_total_, is obtained by integrating Eq. (3) from zero depth to *h*_max_,

4 $${W}_{{\rm{total}}}={\int }_{0}^{{h}_{{\rm{\max }}}}P{\rm{d}}h=\frac{{k}_{1}{h}_{{\rm{\max }}}^{n+1}}{n+1}$$

The unloading curve is described by the following expression^46^,

5 $$P={k}_{2}{(h-{h}_{f})}^{m}$$

where *k*_2_ is the unloading curve constant and *m* is the unloading exponent. *W*_elastic_ is obtained by integrating Eq. (5) from *h*_f_ to *h*_max_,

6 $${W}_{{\rm{elastic}}}={\int }_{{h}_{{\rm{f}}}}^{{h}_{{\rm{\max }}}}P{\rm{d}}h=\frac{{k}_{2}{({h}_{{\rm{\max }}}-{h}_{{\rm{f}}})}^{m+1}}{m+1}$$

Bend tests were performed on bar shaped samples with 6 mm length, 0.6 mm width and 1 mm thickness that were bent around mandrels of different radii at room temperature. The strain to failure can be calculated from *ε* = *h*/2 *R*, where *R* is the neutral radius of the bend sample and *h* is the sample’s thickness. The morphology of fracture surface was observed using an OM.

## Results and Discussion

**Figure 3:**
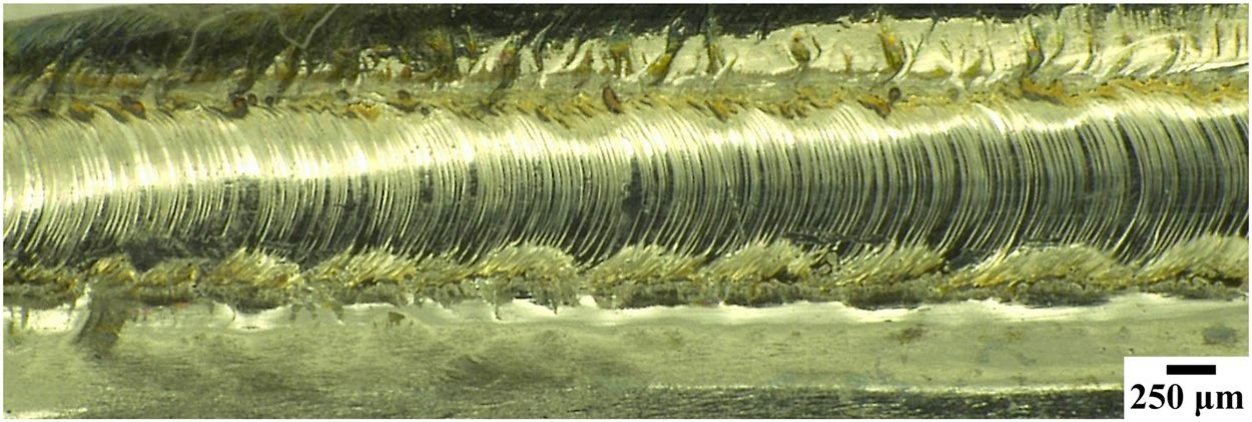
Morphology of bead-on-plate of Pd_43_Cu_27_Ni_10_P_20_ bulk metallic glass obtained by the pulsed laser welding method under 1050 W laser beam power.

**Figure 4:**
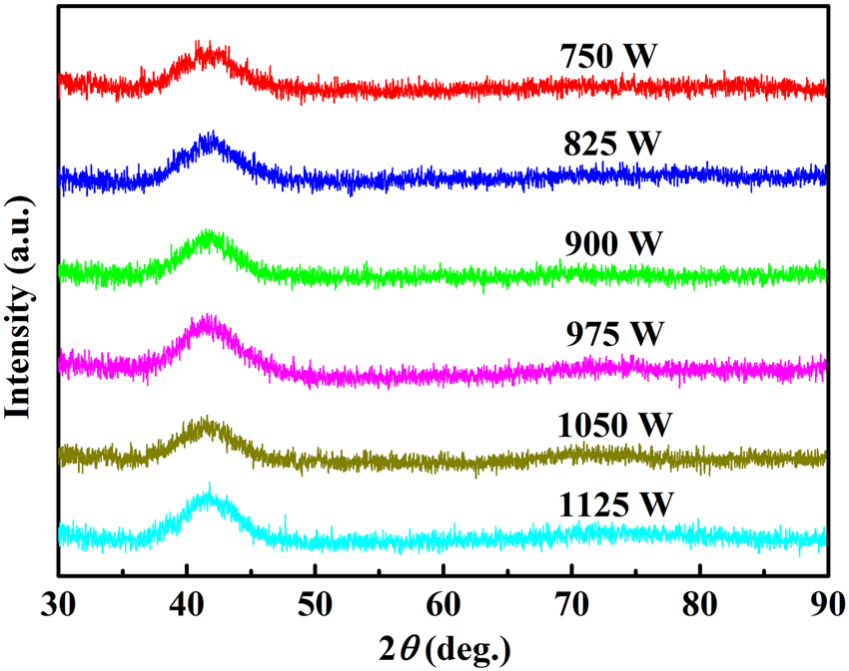
X-ray diffraction pattern of the cross-section of bead-on-plate of Pd_43_Cu_27_Ni_10_P_20_ bulk metallic glass using the pulsed laser welding method with various powers.

The morphology of the weld seams of Pd_43_Cu_27_Ni_10_P_20_ BMG after BOP experiments processed in air was examined by an OM (Fig. 3). The appearance of BOP of Pd_43_Cu_27_Ni_10_P_20_ BMG obtained by the pulsed laser welding technique is uniform, does not exhibit porous and no visible oxidation can be detected. The cross-section of the weld seam was characterized using XRD (Fig. 4). For all considered processing parameters a weld was achieved which resulted in a broad halo, typical for an amorphous sample. This finding implies that the processing conditions and particularly cooling rate of the pulsed laser beam welding method are sufficient to avoid crystallization of Pd_43_Cu_27_Ni_10_P_20_ BMG.

**Figure 5:**
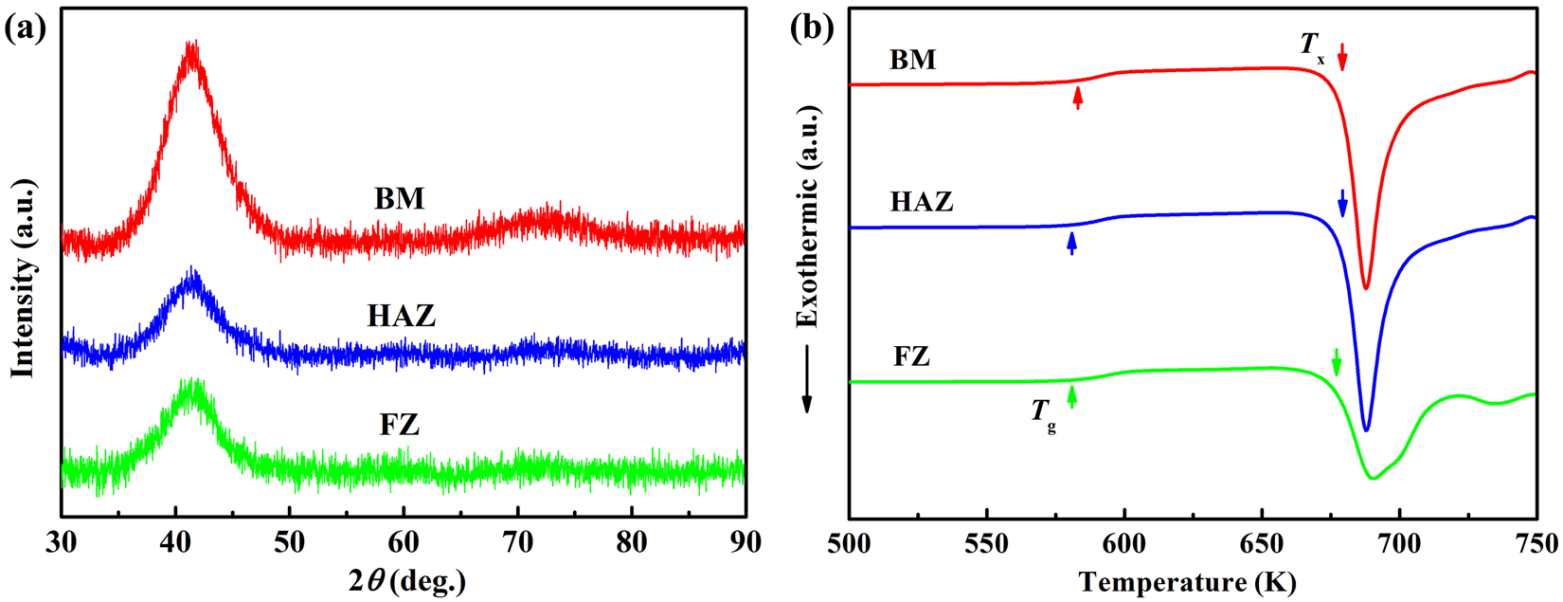
X-ray diffraction patterns (**a**) and differential scanning calorimetry thermograms (**b**) of the base material (BM), heat-affected zone (HAZ) and fusion zone (FZ) in butt joint of Pd_43_Cu_27_Ni_10_P_20_ bulk metallic glass under laser beam power of 1050 W.

Butt welding of Pd_43_Cu_27_Ni_10_P_20_ BMG was carried out using the pulsed laser beam welding technique under 1050 W laser beam power. No visible defects were observed in the butt joint, suggesting a metallurgical bond. XRD patterns of FZ, HAZ and BM are shown in Fig. 5a, indicating the existence of a completely amorphous joint. To further confirm that the butt joint of Pd_43_Cu_27_Ni_10_P_20_ BMG does not contain any crystallinity, a comparison of the DSC traces of the FZ, HAZ and BM with 20 Kmin^−1^ was carried out (Fig. 5b). DSC curves exhibit the similar glass transition temperature, crystallization temperature and heat of crystallization of the different zones in the butt joint of Pd_43_Cu_27_Ni_10_P_20_ BMG. The measured glass transition temperature (*T*_g_) of the FZ and HAZ samples was 582 K and 581 K, respectively. The measured onset crystallization temperature (*T*_x_) of the FZ and HAZ samples was 676 K and 679 K, respectively. The measured heat of crystallization (Δ*H*) of the FZ and HAZ samples was both 76 Jg^−1^. Such values are essentially the same as the base material (*T*_g_ = 580 K, *T*_x_ = 679 K and Δ*H* = 77 Jg^−1^), suggesting that all parts are fully amorphous.

**Figure 6:**
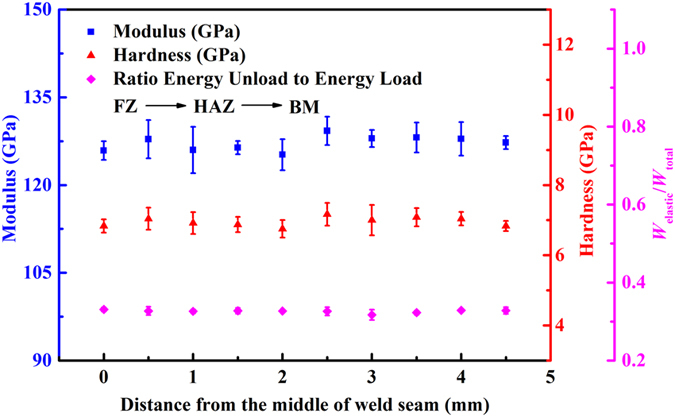
Indentation modulus, hardness and *W*_elastic_/*W*_total_, vs. the distance from the middle of weld seam, plot for the butt joint of Pd_43_Cu_27_Ni_10_P_20_ bulk metallic glass obtained by the pulsed laser welding method.

To study the mechanical properties of the butt joint of Pd_43_Cu_27_Ni_10_P_20_ BMG, nanoindentation tests were carried out from the middle of the weld seam to the BM along the direction perpendicular to the weld seam. Figure 6 shows the variation of indentation modulus, hardness, and *W*_elastic_/*W*_total_ (*W*_total_ = *W*_elastic_ + *W*_plastic_), as a function of the distance from the middle of the weld seam. Indentation energies are useful parameters in analyzing the mechanical behavior of materials, and *W*_elastic_/*W*_total_ was linked to the material’s deformation recovery capability and the initial unloading stiffness^47^. The ratio energy unload to energy load values of FZ, HAZ and BM in the butt joint of Pd_43_Cu_27_Ni_10_P_20_ BMG were essentially identical. Similarly the indentation moduli and hardness of the FZ, HAZ and BM in the weld having amorphous structures did not reveal significant differences. This indicates that the material properties of the FZ and the HAZ are comparable to the base material.

**Figure 7:**
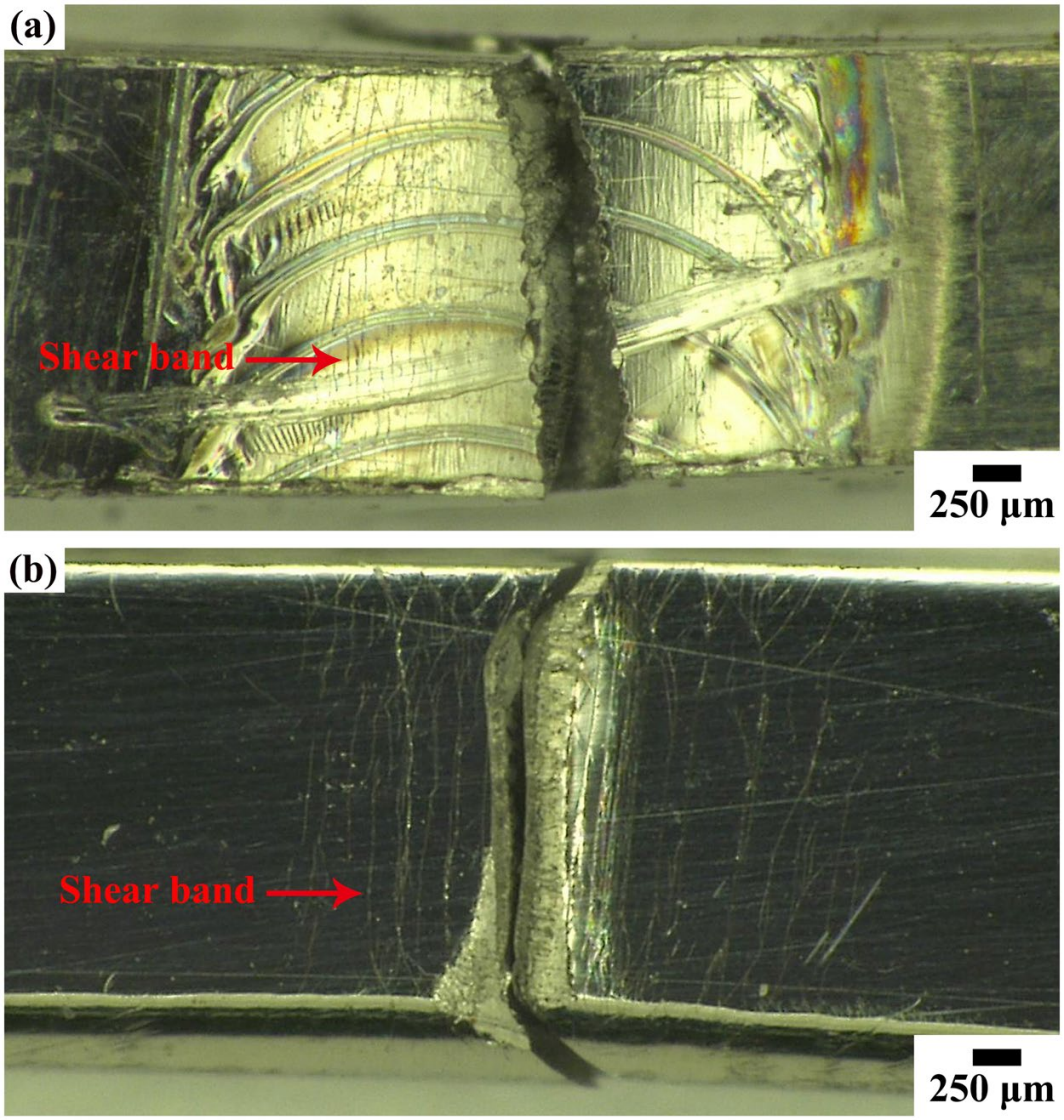
Fracture morphology after bend tests of Pd_43_Cu_27_Ni_10_P_20_ bulk metallic glass: (**a**) weld joint; (**b**) base material.

A powerful test to determine mechanical property of the weld joint is bend test^48–51^. Butt joint and BM samples of Pd_43_Cu_27_Ni_10_P_20_ BMG exhibited significant bending ductility with a failure bending strain of about 7.5% for samples of 1 mm thickness. These numbers are comparable with highest reported bending strains for this alloy^50^. The micrographs of fractures of the bend samples are shown in Fig. 7. Typical vein patterns as found in ductile fracture, were observed over the entire fracture surface of BM sample of Pd_43_Cu_27_Ni_10_P_20_ BMG (Fig. 7b). The fracture position of the butt joint sample is located at the center of the weld seam (Fig. 7a), and the microstructures show multiple shear bands formation with shear band spacing of approximately 25 μm and 60 μm for the butt joint and BM samples, respectively. This confirms that the butt joint of Pd-based BMG possesses the high ductility.

## Summary

We used the pulsed laser beam welding method to join Pd_43_Cu_27_Ni_10_P_20_ BMG. For a range of welding parameters completely amorphous welds can be achieved as quantified by XRD and thermal analysis. The mechanical properties of the weld are comparable with that of the bulk material suggesting that pulsed laser beam welding is a versatile method to join BMGs. For example, it can be used for additive manufacturing to fabricate on demand geometries with superb mechanical properties.

## References

[1] Hofmann DC (2016). Castable bulk metallic glass strain wave gears: Towards decreasing the cost of high-performance robotics. Sci. Rep.

[2] Gludovatz B, Granata D, Thurston KVS, Löffler JF, Ritchie RO (2017). On the understanding of the effects of sample size on the variability in fracture toughness of bulk metallic glasses. Acta Mater..

[3] Denis P (2017). Rejuvenation decreases shear band sliding velocity in Pt-based metallic glasses. Mater. Sci. Eng. A.

[4] Ma E, Ding J (2016). Tailoring structural inhomogeneities in metallic glasses to enable tensile ductility at room temperature. Mater. Today.

[5] Ketov SV (2015). Rejuvenation of metallic glasses by non-affine thermal strain. Nature.

[6] Chen DZ (2015). Fractal atomic-level percolation in metallic glasses. Science.

[7] Huang YJ (2016). Liquid-solid joining of bulk metallic glasses. Sci. Rep.

[8] Kawamura Y, Shoji T, Ohno Y (2003). Welding technologies of bulk metallic glasses. J. Non-Cryst. Solids.

[9] Tariq NH, Iqbal M, Shaikh MA, Akhter JI, Ahmad M (2008). Evolution of microstructure and non-equilibrium phases in electron beam treated Zr_55_Cu_30_Al_10_Ni_5_ bulk amorphous alloy. J. Alloys Compd..

[10] Kagao S, Kawamura Y, Ohno Y (2004). Electron-beam welding of Zr-based bulk metallic glasses. Mater. Sci. Eng. A.

[11] Wang HS, Wu JY, Liu YT (2016). Effect of the volume fraction of the *ex-situ* reinforced Ta additions on the microstructure and properties of laser-welded Zr-based bulk metallic glass composites. Intermetallics.

[12] Pilarczyk W, Starczewska O, Lukowiec D (2015). Nanoindentation characteristic of Fe-based bulk metallic glass laser weld. Phys. Status Solidi. B.

[13] Kim JH, Shin SY, Lee CH (2005). Characterization of the gas tungsten arc welded Cu_54_Ni_6_Zr_22_Ti_18_ bulk metallic glass weld. Mater. Trans..

[14] Kawamura Y (2004). Liquid phase and supercooled liquid phase welding of bulk metallic glasses. Mater. Sci. Eng. A.

[15] Zhou YZ, Zhang QS, He GH, Guo JD (2003). Connection of bulk amorphous alloy Zr_55_Al_10_Ni_5_Cu_30_ by high current density electropulsing. Mater. Lett..

[16] Jamili-Shirvan (2016). Microstructure characterization and mechanical properties of Ti-based bulk metallic glass joints prepared with friction stir spot welding process. Mater. Des.

[17] Kawamura Y, Ohno Y (2001). Superplastic bonding of bulk metallic glasses using friction. Scripta Mater..

[18] Kawamura Y, Ohno Y, Chiba A (2002). Development of welding technologies in bulk metallic glasses. Mater. Sci. Forum.

[19] Zhu ZQ, Wang YJ, Zhang YF (2016). Preparation and study on the properties of bulk amorphous alloy Fe78Si9B13 by ultrasonic welding. J. Optoelectron. Adv. Mater..

[20] Kim J (2014). Weldability of Cu_54_Zr_22_Ti_18_Ni_6_ bulk metallic glass by ultrasonic welding processing. Mater. Lett..

[21] Wang JG (2014). Diffusion bonding of a Zr-based metallic glass in its supercooled liquid region. Intermetallics.

[22] Kawamura Y, Ohno Y (2001). Spark welding of Zr_55_Al_10_Ni_5_Cu_30_ bulk metallic glasses. Scripta Mater..

[23] Guo SF (2016). Microstructure and tensile behavior of small scale resistance spot welded sandwich bulk metallic glasses. J. Non-Cryst. Solids.

[24] Chen W, Liu Z, Schroers J (2014). Joining of bulk metallic glasses in air. Acta Mater..

[25] Kim JH (2007). Pulsed Nd: YAG laser welding of Cu_54_Ni_6_Zr_22_Ti_18_ bulk metallic glass. Mater. Sci. Eng. A.

[26] Kim JH, Lee DM, Shin SY, Lee CH (2006). Phase evolution in Cu_54_Ni_6_Zr_22_Ti_18_ bulk metallic glass Nd: YAG laser weld. Mater. Sci. Eng. A.

[27] Wang HS, Chen HG, Jang JSC, Chiou MS (2010). Combination of a Nd: YAG laser and a liquid cooling device to (Zr_53_Cu_30_Ni_9_Al_8_)Si_0.5_ bulk metallic glass welding. Mater. Sci. Eng. A.

[28] Liu Y (2012). Saffman-Taylor fingering in nanosecond pulse laser ablating bulk metallic glass in water. Intermetallics.

[29] Wang H, Chen H, Jang JS (2010). Microstructure evolution in Nd: YAG laser-welded (Zr_53_Cu_30_Ni_9_Al_8_)Si_0.5_ bulk metallic glass alloy. J. Alloys Compd..

[30] Xia C, Xing L, Long WY, Li ZY, Li Y (2009). Calculation of crystallization start line for Zr_48_Cu_45_Al_7_ bulk metallic glass at a high heating and cooling rate. J. Alloys Compd..

[31] Kawahito Y (2008). High-power fiber laser welding and its application to metallic glass Zr_55_Al_10_Ni_5_Cu_30_. Mater. Sci. Eng. B.

[32] Panwisawas C (2017). Keyhole formation and thermal fluid flow-induced porosity during laser fusion welding in titanium alloys: Experimental and modelling. Acta Mater..

[33] Kumar N, Mukherjee M, Bandyopadhyay A (2017). Comparative study of pulsed Nd: YAG laser welding of AISI 304 and AISI 316 stainless steels. Opt. Laser Technol..

[34] Wang G, Huang YJ, Shagiev M, Shen J (2012). Laser welding of Ti_40_Zr_25_Ni_3_Cu_12_Be_20_ bulk metallic glass. Mater. Sci. Eng. A.

[35] Li B (2006). Laser welding of Zr_45_Cu_48_A_l7_ bulk glassy alloy. J. Alloys Compd..

[36] Schroers J (2008). On the formability of bulk metallic glass in its supercooled liquid state. Acta Mater..

[37] Kim SY (2015). Imprinting bulk amorphous alloy at room temperatue. Sci. Rep.

[38] Yiu P, Hsueh CH, Shek CH (2016). Electroplastic forming in a Fe-based metallic glass ribbon. J. Alloys Compd..

[39] Zhang N, Srivastava AP, Browne DJ, Gilchrist MD (2016). Performance of nickel and bulk metallic glass as tool inserts for the microinjection molding of polymeric microfluidic devices. J. Mater. Process. Technol..

[40] Chmelíčková, H. & Šebestová, H. Nd YAG Laser. (ed. Dumitras, D. C.) 41–58 (In Tech, 2012).

[41] Oliver WC, Pharr GM (2004). Measurement of hardness and elastic modulus by instrumented indentation: Advances in understanding and refinements to methodology. J. Mater. Res..

[42] Datye A (2016). Extraction of Anisotropic Mechanical Properties From Nanoindentation of SiC-6H Single Crystals. ASME. J. Appl. Mech..

[43] Datye A, Lin HT (2017). Energy analysis of spherical and Berkovich indentation contact damage in commercial polycrystalline silicon carbide. Ceram. Int..

[44] Rodríguez M, Molina-Aldareguía JM, González C, LLorca J (2012). Determination of the mechanical properties of amorphous materials through instrumented nanoindentation. Acta Mater..

[45] Sakai M (1999). The meyer hardness: A measure for plasticity?. J. Mater. Res..

[46] Oliver WC, Pharr GM (1992). An improved technique for determining hardness and elastic modulus using load and displacement sensing indentation experiments. J. Mater. Res..

[47] Alao AR, Yin L (2016). Assessment of elasticity, plasticity and resistance to machining-induced damage of porous pre-sintered zirconia using nanoindentation techniques. J. Mater. Sci. Technol..

[48] Conner RD, Johnson WL, Paton NE, Nix WD (2003). Shear bands and cracking of metallic glass plates in bending. J. Appl. Phys..

[49] Kumar G, Rector D, Conner RD, Schroers J (2009). Embrittlement of Zr-based bulk metallic glasses. Acta Mater..

[50] Kumar G, Prades-Rodel S, Blatter A, Schroers J (2011). Unusual brittle behavior of Pd-based bulk metallic glass. Scripta Mater..

[51] Kumar G, Neibecker P, Yanhui L, Schroers J (2013). Critical Fictive Temperature for ductility in metallic glasses. Nat. Commun..

